# Altered functional connectivity in posttraumatic stress disorder with versus without comorbid major depressive disorder: a resting state fMRI study

**DOI:** 10.12688/f1000research.2-289.v2

**Published:** 2014-04-16

**Authors:** Mitzy Kennis, Arthur R. Rademaker, Sanne J.H. van Rooij, René S. Kahn, Elbert Geuze

**Affiliations:** 1 Research Centre-Military Mental Healthcare, Ministry of Defence, 3584 CX Utrecht, Netherlands; 2 Brain Center Rudolph Magnus, University Medical Center Utrecht, 3584 CX Utrecht, Netherlands

## Abstract

Posttraumatic stress disorder (PTSD) is an anxiety disorder that is often diagnosed with comorbid depressive disorder. Therefore, neuroimaging studies investigating PTSD typically include both patients with and without comorbid depression. Differences in activity of the anterior cingulate cortex (ACC) and insula have been shown to differentiate PTSD patients with and without major depressive disorder (MDD). Whether or not comorbid MDD affects resting state functional connectivity of PTSD patients has not been investigated to our knowledge. Here, resting state functional connectivity of PTSD patients with (PTSD+MDD; n=27) and without (PTSD-MDD; n=23) comorbid MDD was investigated. The subgenual ACC and insula were investigated as seed regions. Connectivity between the subgenual ACC and perigenual parts of the ACC was increased in PTSD+MDD versus PTSD-MDD, which may reflect the presence of depressive specific symptoms such as rumination. Functional connectivity of the subgenual ACC with the thalamus was reduced, potentially related to more severe deficits in executive functioning in the PTSD+MDD group versus the PTSD-MDD group. In addition, the PTSD+MDD group showed reduced functional connectivity of the insula with the hippocampus compared to the PTSD-MDD group. However, this cluster was no longer significantly different when PTSD patients that were using medication were excluded from analyses. Thus, resting state functional connectivity of the subgenual ACC can distinguish PTSD+MDD from PTSD-MDD, and this may therefore be used as a neurobiological marker for comorbid MDD in the presence of PTSD. As PTSD+MDD are more treatment resistant, these findings can also guide treatment development, for example by targeting the subgenual ACC network with treatment.

## Introduction

Posttraumatic stress disorder (PTSD) is an anxiety disorder that can develop after a traumatic event. It is characterized by re-experiencing the traumatic event, avoidance of trauma reminders and emotional numbing symptoms, and increased arousal ^1^. PTSD frequently co-occurs with other Axis I psychiatric disorders, such as major depressive disorder (MDD ^2^). Patients with both PTSD and depression were found to have more psychological distress and are also more treatment resistant than patients with PTSD or depression alone ^3– 5^. Studies have demonstrated that comorbidity between mood and anxiety disorders increases risk for cardiovascular disease, autoimmune diseases and mortality ^6, 7^. In addition, depressive symptom severity and comorbidity of MDD are related to poorer executive functioning in PTSD ^8, 9^. In order to better prevent, diagnose or treat these disorders it is of importance to determine biological overlap and differences between mood and anxiety disorders, and also the effect of comorbidity. About 48% of PTSD patients were found to have comorbid MDD in a large national survey in the United States ^2^. Therefore, studies investigating the neurobiology of PTSD often comprise patients with and without comorbid MDD. Neuroimaging studies have demonstrated dysfunction of similar brain regions in both PTSD and MDD. That is, PTSD and MDD are both associated with alterations in structure and function of the medial prefrontal cortex (mPFC), amygdala, insula, and anterior cingulate cortex (ACC ^10– 12^). To what extent comorbid MDD contributes to the reported neurobiological alterations of PTSD is yet to be determined.

Thus far, two neuroimaging studies have directly investigated differences in PTSD patients with and without comorbid MDD. First, reduced activity of the mPFC and amygdala was found in PTSD patients with comorbid MDD versus PTSD patients without MDD, when fearful faces were presented ^13^. Second, during a symptom provocation paradigm PTSD patients with comorbid MDD had decreased activity in the insula, and increased ACC and posterior cingulate cortex (PCC) activation versus PTSD patients without MDD ^14^. In addition, decreased insula activation remained significant after controlling for PTSD severity. One other study has investigated the effects of depressive symptoms in PTSD patients. A positive correlation between depressive symptoms and (para) hippocampal and ventral ACC activity during an emotional memory task was observed in PTSD patients. A fourth fMRI study involving PTSD patients versus both controls and MDD patients found increased activity in several brain areas of PTSD patients including the insula when emotional pictures were presented ^15^.

The four studies discussed above were limited by small sample sizes (8 PTSD-MDD, 8 PTSD+MDD ^13^, 11 PTSD-MDD and 15 PTSD+MDD ^14^, 21 PTSD+MDD and 12 PTSD-MDD ^16^, 16 PTSD and 16 MDD ^15^). In addition, these studies investigated neurobiological alterations during emotional tasks, potentially inducing PTSD (and/or depressive) symptoms. It is expected that PTSD and/or MDD symptom provocation induces an altered state in PTSD with or without MDD, which is reflected by alterations in brain activity. Whether regular functioning of the brain in the absence of symptom-inducing stimuli deviates in PTSD with versus without comorbid MDD remains unclear. To our knowledge, functioning of the brain during resting state, without presenting stimuli or requiring task performance, has not been investigated in PTSD patients with and without comorbid MDD. Thus, the effect of comorbid MDD on brain functioning at baseline of PTSD patients deserves further investigation.

Here, we investigate the effects of comorbid MDD on resting state functional connectivity in PTSD patients. Since the studies described above indicated that functioning of the ACC distinguishes PTSD with and without MDD during emotional tasks ^13, 14, 16^, this brain area was chosen as a region of interest. MDD has been associated with alterations in structure ^17^, function ^18^, structural connectivity ^19^, and reduced resting state functional connectivity ^20– 22^ of the subgenual ACC in particular, which is a subdivision of the ventral ACC. In addition, subgenual ACC activation and cortical thickness have been associated with symptom improvement in PTSD ^23, 24^. Therefore, the subgenual ACC was selected as a more specific region of interest. Second, alterations in activation of the insula also differed between PTSD patients with and without PTSD, even when controlling for PTSD severity ^14^. Furthermore, insula activation distinguished PTSD patients from MDD patients ^15^. Alterations in structure ^10, 25^, function ^10, 26^, and resting state functional connectivity ^27, 28^ have been reported in PTSD patients and MDD patients respectively. Thus, the insula was chosen as a second region of interest. As increased ACC activity was found in PTSD with comorbid MDD, as well as a positive correlation of ACC activity with depressive symptoms, we hypothesize that functional connectivity of the subgenual ACC is increased in PTSD with versus without comorbid MDD. Since insula activity is increased in PTSD versus MDD and insula activity was reduced in PTSD with comorbid MDD versus PTSD without MDD, we expected to find lower insula functional connectivity in PTSD with MDD as compared to PTSD without MDD. In summary, in order to provide more insights into the potential effects of MDD on the neurobiology of PTSD, the present study examined the effects of comorbid MDD on subgenual ACC and insula resting state functional connectivity in PTSD patients.

## Methods

### Participants

In total, 30 male veterans with PTSD with comorbid MDD (PTSD+MDD, mean age 34.2 ± 8.5), and 25 male veterans with PTSD without comorbid MDD (PTSD-MDD, mean age 37.4 ± 10.1) were included in this study. All patients were recruited from the Military Mental Health Care Center, the Netherlands. Patients were included after a clinician (psychologist or psychiatrist) diagnosed PTSD with or without MDD. PTSD and MDD diagnoses were confirmed using the Clinician Administered PTSD scale (CAPS ^29^) and the Structural Clinical interview for DSM-IV (SCID ^30^). A clinician, a trained PhD student or a trained research assistant administered the interviews. Training included a CAPS training, and additionally observing at least five interviews, and performing at least five interviews under supervision of an experienced clinician. Several patients were medication naive (PTSD+MDD; n=15, PTSD-MDD; n=13), some patients were currently taking antidepressants (e.g. selective serotonin reuptake inhibitors; PTSD+MDD; n=4, PTSD-MDD; n=5), and some patients used benzodiazepines (PTSD+MDD; n=4, PTSD-MDD; n=1), or both antidepressants and benzodiazepines (PTSD+MDD; n=2, PTSD-MDD; n=2). One patient from the PTSD+MDD group used both antipsychotics and antidepressants. Most of the veterans had been deployed to Afghanistan (n=28) and to the former Yugoslavia (n=10). After receiving a complete written and verbal description of the study, all participants gave informed consent. Participants received financial compensation of €250 for their participation. The Medical Ethical Committee of the UMC Utrecht approved the study (protocol number NL29550.041.09), and the study was performed in accordance with the Declaration of Helsinki ^31^.

### Data acquisition

Functional and structural images were obtained using a 3.0 Tesla magnetic resonance imaging scanner (Philips Medical System, Best, the Netherlands). Before the resting state scan, a ten minute T1-weighted high-resolution image (TR = 10 ms TE = 4.6 ms flip angle 8, 200 slices sagittal orientation, FOV 240 × 240 × 160, 304 × 299 matrix) was acquired. This image was utilized for co-registration and segmentation purposes and also allowed the participants to adapt to the scanner environment. During the nine minute resting state scan participants were asked to relax, to let their mind wander and to focus on a fixation cross. Three hundred and twenty T2* echoplanar interleaved images were collected (TR = 1600 ms, TE = 23 ms, flip angle = 72.5°, 30 transverse slices, FOV 256 × 208 × 120, 64 × 51 matrix).

### Image analyses

Pre-processing was conducted with SPM5 ( http://www.fil.ion.ucl.ac.uk/spm/software/spm5/), which included slice-timing correction, realignment, co-registration with the anatomical scan, normalization, and spatial smoothing (8 mm FWHM). Five participants (2 PTSD+MDD, 3 PTSD-MDD) were excluded due to excessive motion (more than 2 mm displacement in any direction (x, y or z) or 2 degrees rotation (pitch, roll or yaw)).

The Data Processing Assistant for Resting-State fMRI (DPARSF) was utilized for further analyses (restfmri.net ^32^), which is based on MRIcroN ( http://www.mricro.com), SPM5 ( http://www.fil.ion.ucl.ac.uk/spm/software/spm5/), and the Resting-State fMRI Data Analysis Toolkit ^32^. Resting state images were band-pass filtered (0.08–0.01 Hz) to reduce low-frequency drift and high-frequency noise, and detrended to correct for general signal drift. In order to correct for physiological processes and motion, the motion parameters from the realignment step, mean global signal, white matter signal, and cerebral spinal fluid signal were included as covariates in the analysis. In addition, motion scrubbing was applied to scans that surrounded a minimum of 0.5 mm frame displacement (one scan before displacement, two scans after displacement), using nearest neighbour interpolation ^33^. A minimum of approximately 5 minutes of resting state (183 unscrubbed resting state images) was set as a required threshold for correct scrubbing. One participant was excluded due to excessive scrubbing, resulting in the following groups: 27 PTSD+MDD, and 22 PTSD-MDD.

### Functional connectivity analysis

For the subgenual ACC two spherical seeds (left and right, 3.5 mm radius) were created around two seed point coordinates, as previously described by Kelly *et al.* (2009) ^34^. The anterior insula seed was created from two distinct anterior insula subdivisions that were described as the insula regions involved in emotion and cognition, as reported by Kelly *et al.* (2012) ^35^. The mean time series for each of those seeds was extracted for all individuals and correlated with the time series of every voxel in the brain in order to create functional connectivity maps. These correlation maps were normalized using Fishers z-transform, resulting in a z-map for each ACC network per participant. The individual z-maps were used for second-level group analysis (full factorial design, SPM). A general effect of group (F-test) was investigated to determine group differences within the positive and negative network of the seed pairs.

Cluster-level multiple comparison correction was applied according to Gaussian Random Field theory ^36^. A height threshold of p<0.001 was applied and combined with a cluster threshold extent that corresponds to a corrected p<0.05 (as determined with 1000 Monte Carlo simulations using Alphasim, implemented in the REST toolbox).

In addition, functional connectivity values (z-values) were extracted from the peak voxels of clusters of significant differences in order to perform post-hoc correlations with PTSD and MDD symptom severity. Post-hoc correlation analyses were performed including the total CAPS score and the signal extracted from the peaks of clusters of significant connectivity differences, in order to assess whether the results are related to PTSD severity. In addition, the relation of positive affect (PA) score from the mood and anxiety questionnaire (MASQ ^37^), which has been reported to reflect a core feature of MDD ^38^, to the functional connectivity of the peak of the clusters of significant difference was assessed. Subsequently, correlations between whole brain functional connectivity and CAPS and inverse PA scores were calculated respectively. Finally, we performed a post-hoc analysis on a subsample of medication naive patients and patients that occasionally used benzodiazepines, but had not taken benzodiazepines at least 48 hours prior to scanning.

## Results

### Participants

Groups did not differ significantly in age, handedness, the number of times they were deployed, the time since their last deployment, and educational level as measured with the international standard classification of education (ISCED ^39^). The PTSD+MDD group differed from the PTSD-MDD group in total PTSD severity (CAPS score; p=0.008), which appeared to be largely driven by differences in avoidance and emotional numbing symptom scores (cluster C; p=0.001). In addition, the PTSD+MDD group had lower PA scores versus the PTSD-MDD group (p=0.012), while negative affect and somatic anxiety did not differ between groups. In the PTSD+MDD group 10 patients were diagnosed with a comorbid anxiety disorder (n=10), and one patient had a comorbid somatoform disorder. In the PTSD-MDD group seven patients met the current diagnostic criteria for a comorbid anxiety disorder, one patient had a somatoform disorder only, and one patient was diagnosed with both a comorbid anxiety and somatoform disorder. An overview of demographical and clinical data is presented in Table 1.

**Table 1.** Demographic and clinical characteristics of the PTSD+MDD and the PTSD-MDD group.

Measure	PTSD + MDD (mean ± SD)	PTSD - MDD (mean ± SD)	df	Sig. (two-tailed)
N	27	22		
Age (range 21–57)	37.41 (±10.12)	33.87 (±8.43)	47	0.239
Education (ISCED level)	4.00 (±1.20)	3.65 (±1.23)	46	0.311
Handedness (Right/Left/Ambidexter)	(21/4/2)	(20/0/2)	2	0.169
Number of times deployed (range 1–15)	2.16 (±1.43)	3.18 (±4.22)	45	0.898
Time since last deployment (years)	8.00 (±8.537)	7.05 (±8.72)	45	0.706
Country of last deployment				
Afghanistan	13	17		
Former Yugoslavia	6	3		
Other	8	5		
CAPS total score	75.15 (±12.45)	65.09 (±12.87)	47	0.008*
Cluster B	22.67 (±5.61)	22.64 (±5.43)	47	0.985
Cluster C	27.48 (±8.76)	18.59 (±8.30)	47	0.001*
Cluster D	25.00 (±4.47)	23.86 (±4.97)	47	0.404
Negative Affect (MASQ)	52.12 (±14.91)	46.00 (±10.50)	42	0.130
Positive Affect (MASQ)	40.87 (±15.80)	51.70 (±10.50)	42	0.012*
Somatic Anxiety (MASQ)	44.75 (±13.52)	41.50 (±10.91)	42	0.392
Current comorbid disorder (SCID)				
Major depressive disorder	27	-		
Anxiety disorder	10	7		
Anxiety disorder & somatoform disorder	-	1		
Somatoform disorder	1	1		

*Significant differences between groups; p<0.05

### Functional connectivity

**Figure 1.**
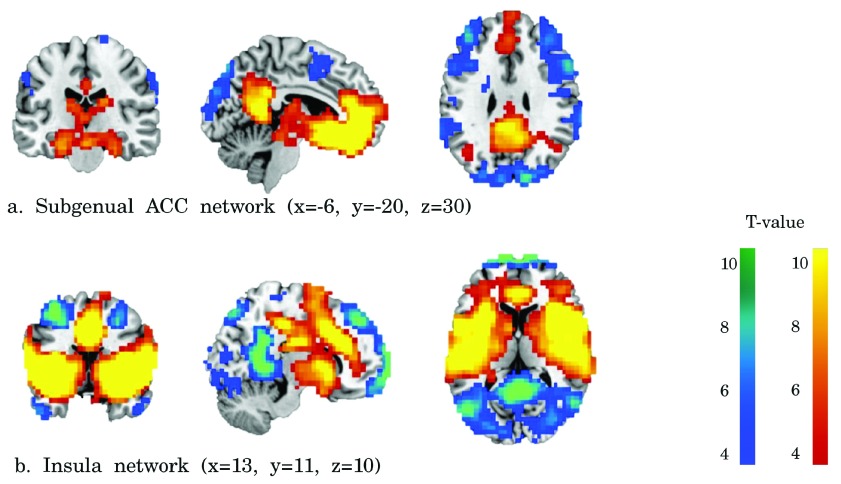
Functional connectivity of the subgenual ACC ( **a**), and insula ( **b**) seeds. Positive connectivity is represented in red-yellow and negative connectivity in blue-green. The effects were FDR corrected p<0.001 for illustrative purposes.

***Spatial connectivity maps.*** Figure 1 shows the positive and negative networks for the bilateral insula and the bilateral subgenual ACC. Positive functional connectivity of the subgenual ACC was found with the ventromedial PFC, temporal regions (including the hippocampus) and a posterior cluster comprising the PCC/precuneus. Positive functional connectivity of the insula was found around the insular lobe, extending into the temporal and parietal lobe. A medial cluster around the dorsal ACC showed positive functional connectivity with the insula.

### Group differences

**Figure 2.**
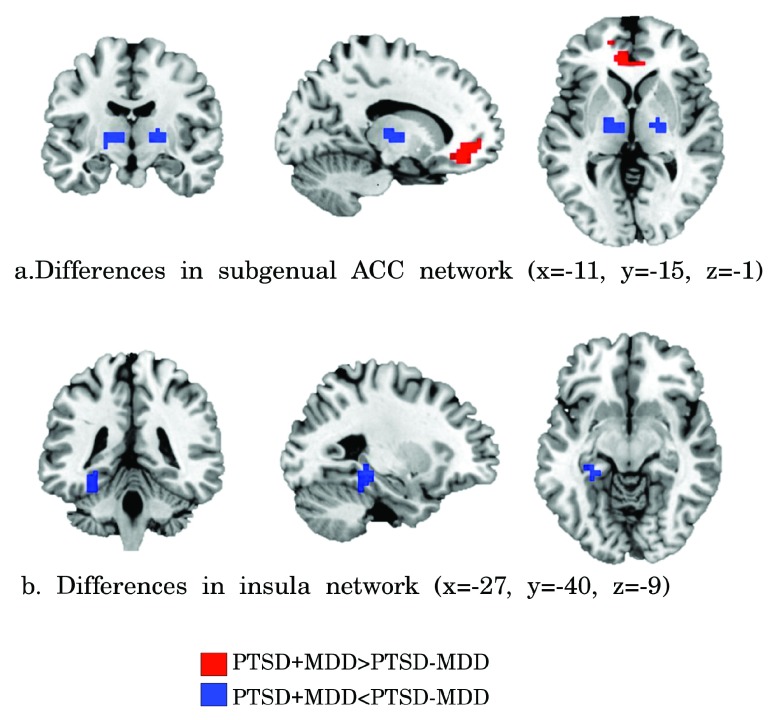
Clusters of significant different functional connectivity of the insula ( **a**) and subgenual ACC ( **b**) seeds. Increased functional connectivity in PTSD+MDD versus PTSD-MDD is shown in red and reduced connectivity in blue (FDR corrected p<0.05).

**Figure 3.**
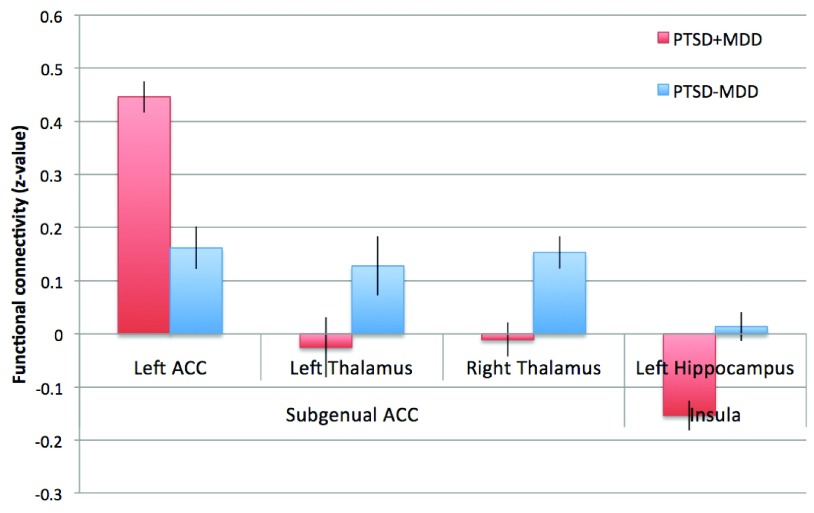
Functional connectivity of peak voxels of significant differences for the subgenual ACC and insula network. Z-values of the peak voxels for the PTSD-MDD group (red) and the PTSD+MDD (blue) group are presented. Error bars represent the standard error of the mean. Z-values of the peak voxels for the PTSD-MDD group (red) and the PTSD+MDD (blue) group are presented.

***Subgenual ACC.*** Reduced functional connectivity of the PTSD+MDD group versus the PTSD-MDD group was found in functional connectivity of the subgenual ACC with the bilateral thalamus (Left thalamus; 29 voxels; peak value F=25.71; peak MNI-coordinates x=-12, y=-16, z=4. Right thalamus; 16 voxels; peak value F=34.37; peak MNI-coordinates x=20, y=-12, z=4). Increased functional connectivity was found between the subgenual ACC and perigenual regions of the ACC (peak in left perigenual ACC; 100 voxels; peak value F=25.71; peak MNI-coordinates x=-12, y=40, z=-4) in the PTSD+MDD group versus the PTSD-MDD group (see Figure 2, Figure 3 and Table 2).

**Table 2.** Peak voxels of significant differences between PTSD+MDD and PTSD-MDD for the subgenual ACC and insula.

Network	Number of voxels (k)	Peak value (F)	MNI coordinates			Brain area
			x	y	z	
*Subgenual ACC*	100	25.71	-12	40	-4	Left Anterior cingulate cortex
	29	23.79	-12	-16	4	Left thalamus
	16	34.37	20	-12	4	Right thalamus
*Insula*	17	19.05	-28	-32	-8	Left hippocampus

***Insula.*** Functional connectivity of the bilateral insula with the left hippocampus (17 voxels; peak value F=19.05; peak MNI-coordinates x=-28, y=-32, z=-8) was reduced in the PTSD+MDD group as compared to the PTSD-MDD group, which showed no functional connectivity between these regions (see Figure 2, Figure 3, and Table 2).

### Post-hoc analyses

**Figure 4.**
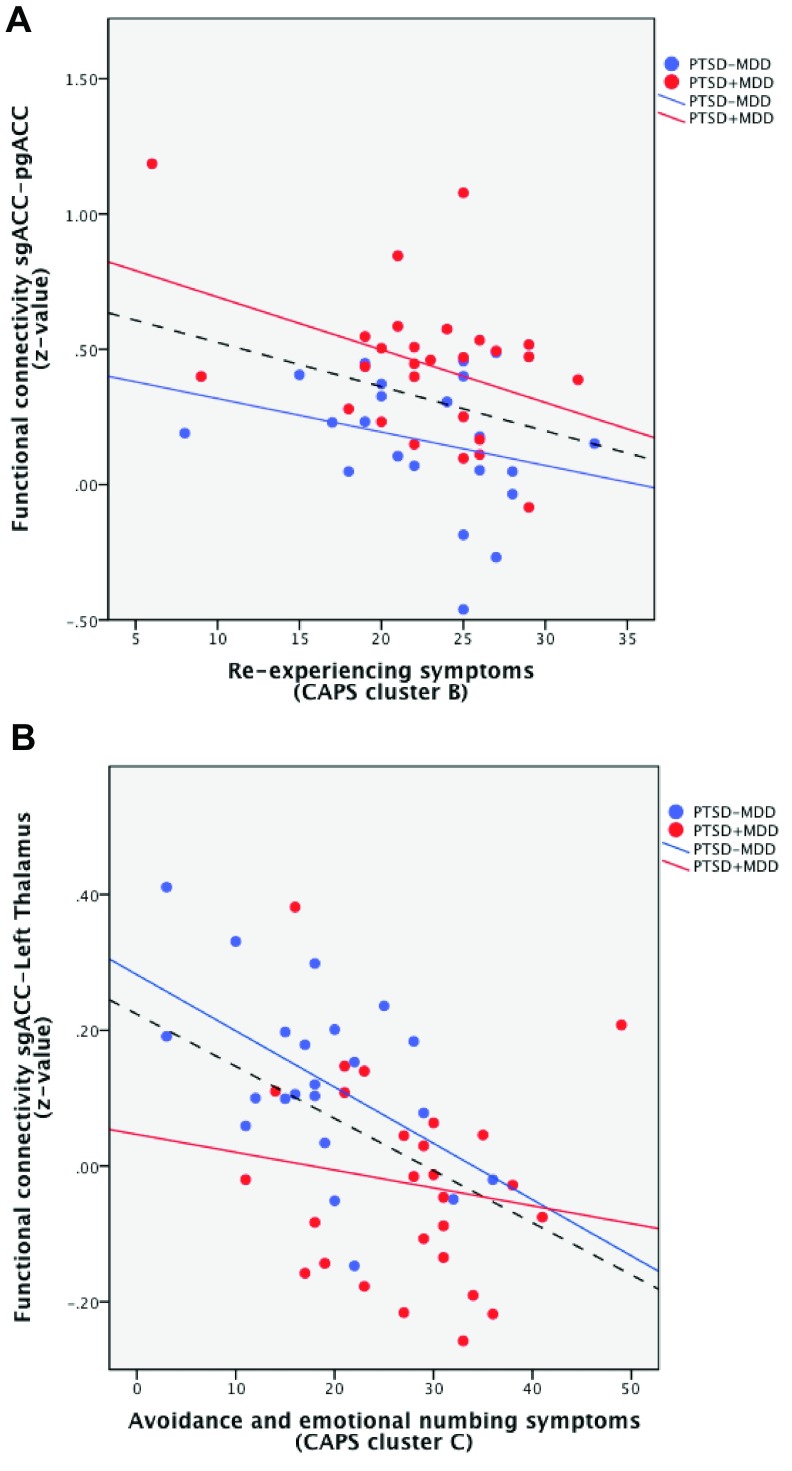
Correlations between CAPS symptom cluster scores and resting state functional connectivity of peak voxels of the significant different clusters within the PTSD+MDD group (red) and the PTSD-MDD group (blue). A correlation line for the whole group is also represented for illustrative purposes (dashed black line). Connectivity of the subgenual ACC with the perigenual ACC correlated with cluster B symptoms (re-experiencing; **4a**). Connectivity of the subgenual ACC with the left thalamus correlated with cluster C symptoms (avoidance and emotional numbing; **4b**). Abbreviations: sgACC: subgenual ACC, pgACC: perigenual ACC.

Post-hoc correlation analyses of the peak voxels of significant functional connectivity difference with CAPS total, CAPS symptom cluster, and inverse PA scores were performed within both groups separately. No significant correlations were found between the peak voxels and total CAPS score and inverse PA scores. Correlations with symptom clusters revealed two significant correlations and these correlations are also represented over all participants for illustrative purposes ( Figure 4). Within the PTSD+MDD group CAPS cluster B scores correlated negatively with connectivity of the subgenual ACC with the peak voxel of significant difference in the perigenual ACC (r = -0.396, p=0.041; Figure 4a). CAPS cluster C scores correlated negatively with connectivity of the subgenual ACC with the peak voxel of significant difference in the left thalamus (r = -0.523, p=0.012) within the PTSD-MDD group ( Figure 4b). No correlations were found between CAPS cluster D scores or inverse PA scores and the peak voxels of difference in connectivity.

**Supplementary Figure 1.**
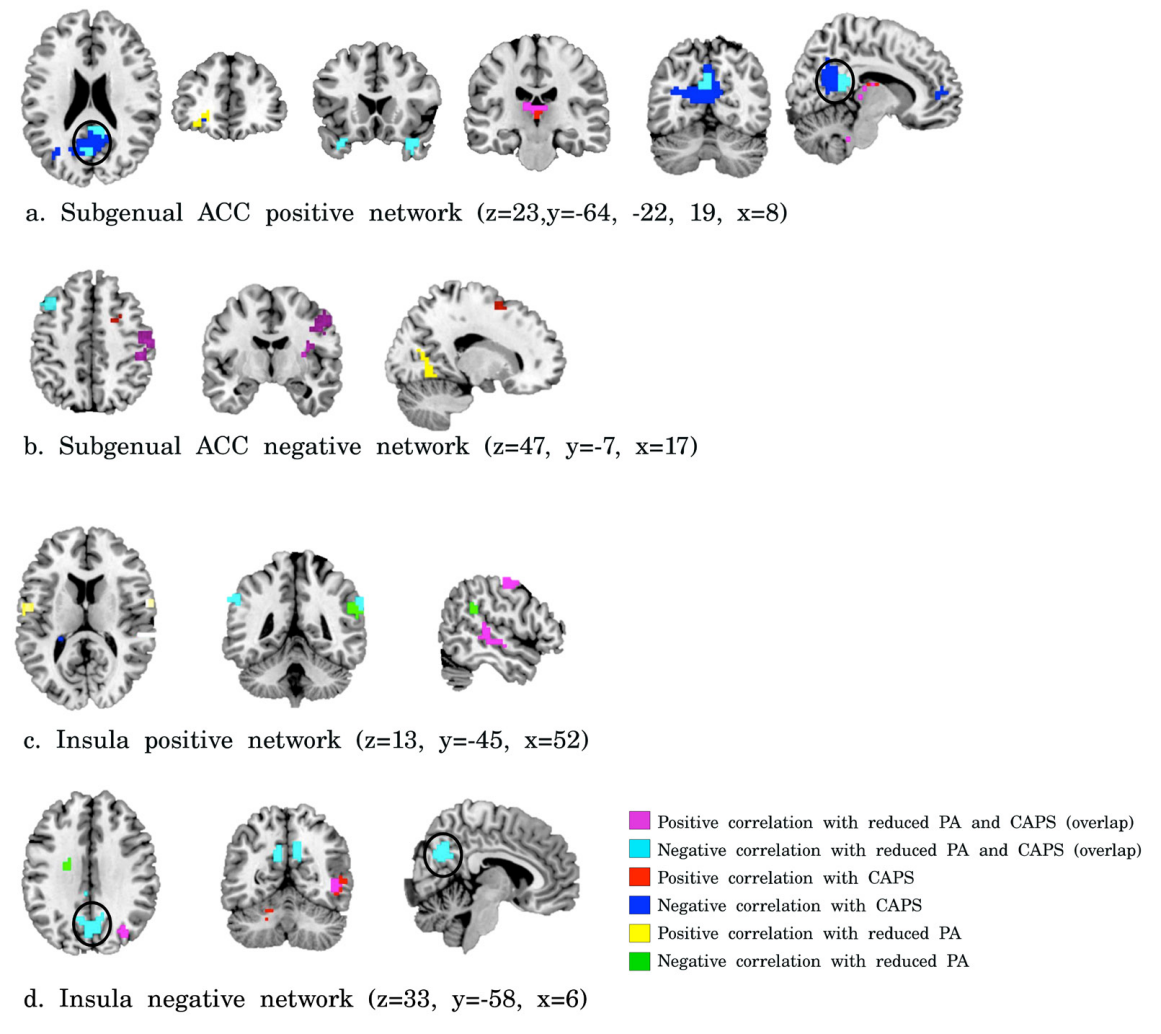
Correlations of PTSD symptom severity and reduced positive affect with subgenual ACC ( **a**, **b**) and insula ( **c**, **d**) functional connectivity. Violet = positive correlation with both CAPS scores and reduced PA, cyan = negative correlations with both CAPS scores and reduced PA, red = positive correlations with CAPS scores, blue = negative correlations with CAPS, yellow = positive correlations with reduced PA, and green = negative correlation with reduced PA (corrected p<0.05).

Exploring the relation of whole brain subgenual ACC connectivity with CAPS and inverse PA scores revealed a negative correlation of CAPS and inverse PA scores with subgenual ACC-PCC/precuneus connectivity, amongst other regions (see Supplementary Figure S1). In addition, a negative correlation was found between CAPS and inverse PA scores and negative functional connectivity of the insula with the PCC/precuneus (see Supplementary Figure S1).

Finally, when PTSD patients that were taking medication were excluded from analyses (PTSD+MDD n = 20, PTSD-MDD n = 15) similar clusters of significant differences for the subgenual ACC network were found. The cluster of significant differences in functional connectivity between the hippocampus and insula was no longer significant in this subsample.

fMRI data of PTSD patients with and without comorbid Major Depressive Disorder Patients were male veterans recruited from the Military Mental Health Care Center, the Netherlands. MDD_diagnosis = current depressive episode (1=yes, 0=no), CAPS_TOTAL_B = total scores from CAPS cluster B symptoms, CAPS_TOTAL_C = total scores from CAPS cluster C symptoms, CAPS_TOTAL_D = total scores from CAPS cluster D symptoms, MASQ_NA = negative affect scores, MASQ_PA = positive affect scores, MASQ_SA = somatic anxiety scores, RSFC=resting state functional connectivity, Hip = hippocampus, ACC= anterior cingulate cortex, sg= subgenual, Thal = thalamus, SSRI = current use of selective serotonin reuptake inhibitor (0=no, 1=yes), BENZO = current use of benzodiazepines (0=no, 1=yes), SARI = current use of serotonin antagonist and reuptake inhibitor (0=no, 1=yes), ISCED = international standard classification of education. Handedness presents right-handedness as 0, left-handedness as 2 and ambidexterity as 3. The code 9999 represents a missing value.

## Discussion

This study compared PTSD patients with and without comorbid depressive disorder and revealed differences in resting state functional connectivity of the subgenual ACC with the perigenual ACC and thalamus, and of the insula with left hippocampus. This study complements previous task-based studies ^13, 14^ by showing that differences in the subgenual ACC and insula between PTSD patients with and without comorbid MDD are already apparent during resting state functional connectivity, in the absence of symptom-inducing stimuli or task performance. Based on these findings, it can be hypothesised that MDD comorbidity in the context of PTSD is related to general alterations in subgenual ACC and insula functioning.

Increased subgenual ACC connectivity with the perigenual ACC was found in PTSD+MDD versus PTSD-MDD, which is in line with neuroimaging studies that have found increased resting state functional connectivity between the subgenual ACC and perigenual ACC in MDD versus controls ^20– 22^. Reduced functional connectivity of ACC regions has been shown in PTSD patients versus controls ^40^. Thus, the current finding of increased connectivity of the subgenual ACC with the perigenual ACC may indeed be a marker of the presence of MDD in the context of PTSD. The perigenual ACC, which is part of the medial PFC, has been related to self-referential processing ^41^, which underlies depressive symptoms such as helplessness, self-reproach and (guilt) rumination ^21, 42^. Increased resting state functional connectivity in the medial PFC (including the perigenual ACC) has been directly related to rumination in MDD ^43^, while decreased functional connectivity with the medial PFC has been related to autobiographical memory recall in PTSD versus controls ^44^. Altered functioning of the medial PFC during self-referential processing tasks has also been found in MDD patients versus controls ^42, 45, 46^ (reduced medial PFC deactivation), and in PTSD versus controls ^47^ (reduced medial PFC activation). Increased subgenual-perigenual ACC connectivity in the PTSD+MDD group versus the PTSD-MDD group could thus reflect a difference in self-referential processing, and potentially reflects symptoms such as rumination. However, this was not directly investigated here, and is subject to further investigation.

A negative correlation between re-experiencing symptoms and functional connectivity of the subgenual ACC and perigenual ACC was found within the PTSD+MDD group (and across all patients). The same pattern was visible in the PTSD-MDD group, although this correlation was not significant. These correlations indicate that stronger functional connectivity between the subgenual ACC and perigenual ACC is related to lower (PTSD-specific) re-experiencing symptoms. This is in line with a previous study describing reduced connectivity in midline structures during autobiographical memory recall in PTSD versus controls ^44^, indicating that the medial PFC can indeed be involved in re-experiencing autobiographical traumatic events. Thus, stronger functional connectivity between the subgenual ACC and perigenual ACC may reflect the presence of MDD, and is also negatively related to (PTSD specific) re-experiencing symptoms.

Connectivity between the thalamus and subgenual ACC was reduced in PTSD+MDD versus PTSD-MDD, which was also reported in previous studies in both depression ^48^ and PTSD ^49^ versus healthy controls. The thalamus is the relay station of the brain ^50^, and can modulate attention and arousal ^51^. Therefore, reduced thalamus-subgenual ACC connectivity may explain the more severe problems with executive function that are prevalent in PTSD with comorbid MDD ^8, 9^. Functional connectivity between the subgenual ACC and thalamus was negatively correlated with avoidance and emotional numbing symptoms in the PTSD-MDD group (and across all participants). Emotional numbing is a shared PTSD and MDD symptom. A weaker connection between the subgenual ACC and thalamus, that was found in PTSD+MDD versus PTSD-MDD, may therefore reflect the presence of depression-related symptoms. Thus, reduced thalamus-subgenual ACC connectivity is a marker for comorbid MDD in the context of PTSD, and also relates to avoidance and emotional numbing symptoms.

Insula connectivity with the hippocampus was reduced in the PTSD+MDD group versus PTSD-MDD. The hippocampus is a brain region that is often associated with PTSD ^11, 52, 53^ and is involved in memory ^54^. Therefore, differences found in connectivity between the insula and hippocampus can be related to more severe difficulties in executive functioning that are prevalent in PTSD+MDD versus PTSD-MDD ^8, 9^. However, the cluster was no longer significant when patients that were taking medication were excluded from analyses. Thus, hippocampus-insula connectivity differences may have been induced by medication use.

In our whole brain post-hoc correlation analysis negative correlations were found between symptom severity scores and subgenual ACC connectivity with the PCC/precuneus (see Supplementary Figure S1a). Specific correlations between CAPS scores and subgenual ACC-PCC/precuneus connectivity were also present, while controlling for inverse PA scores. The medial PFC (including ACC regions) and PCC/precuneus are regions of the default mode network (DMN), which is the network that is active during rest and deactivated during task performance ^55^. DMN functional connectivity has been negatively correlated with general symptom severity in PTSD in previous studies, even when correcting for depression diagnosis ^56^ and depression severity ^57^, which is in line with our results. In addition, a negative correlation was found between symptom severity scores and negative functional connectivity (anticorrelation) between the insula and PCC/precuneus (see Supplementary Figure S1d). Alterations in anticorrelation between the insula network and the DMN has been described in PTSD and depression ^27, 28, 58^. In healthy subjects the insula-PCC/precuneus anticorrelation represents a dynamic equilibrium between engagement of networks during different circumstances, and dysfunctional anticorrelation is thought to underlie attentional problems ^59^. Thus, the negative correlation between symptom severity and anticorrelation between DMN and insula may reflect a disequilibrium between networks and can potentially be related to attentional problems in PTSD patients (with or without comorbid depression).

Unravelling the neurobiological features of MDD and PTSD during rest can provide insights into which specific brain areas could be targeted for effective treatments. For example, tasks, psychotherapy, or brain stimulation methods that alter functional connectivity between the regions with dysfunctional connectivity may be effective ^60, 61^. Future studies should investigate long-term effects of training, transcranial magnetic stimulation, transcranial direct current stimulation, or deep brain stimulation on functional connectivity. In addition, in severe treatment resistant PTSD+MDD surgical treatment may be considered, targeting the regions with altered functional connectivity. The thalamus for example has already been implicated as a target for deep brain stimulation of severe MDD ^62^ and can therefore be a candidate for treatment in PTSD+MDD as well. This is particularly relevant for treatment of PTSD patients with comorbid MDD, since patients with this combination of psychiatric disorders tend to be more treatment resistant.

## Limitations

This study has some limitations. First, no MDD only group or control group was included for analyses in the current study. Thus, this study does not show whether subgenual ACC and insula connectivity differs from patients with MDD only nor does it show if the patients deviate from controls. The current results only give insight in the effects of comorbid MDD in the context of PTSD, and not on general effects of PTSD or MDD. Inclusion of more control groups in future research can provide more insight in the specific effects of PTSD, MDD, and their neurobiological overlap or differences. Second, no validated measure of the severity of all MDD symptoms was included in the study. If MDD severity was measured, it would have been possible to determine common and distinct factors of PTSD symptom severity and MDD symptom severity by including both measures in a single model (as attempted in the Supplementary Figure S1). Here, MDD diagnosis was determined with the SCID, and depressive symptom severity was approximated with the positive affect scale of the MASQ, which is only representative of a subset of symptoms (reduced positive affect). Future studies should investigate the specific effect of MDD symptom severity in the presence of comorbid PTSD, measured with more sensitive and comprehensive instruments.

## Conclusion

This study revealed differences between PTSD+MDD and PTSD-MDD in resting state functional connectivity of the subgenual ACC with the perigenual ACC and bilateral thalamus. Reduced connectivity of the perigenual ACC with the subgenual ACC may be related to specific depressive symptoms, such as rumination. A negative relation was found with PTSD-specific re-experiencing symptoms, indicating that reduced subgenual ACC connectivity with the perigenual ACC is a marker of MDD and negatively related to PTSD-specific symptoms. Increased thalamus connectivity with the subgenual ACC can potentially be related to deficits in executive functioning in PTSD+MDD versus PTSD-MDD. Differences in connectivity of the insula and hippocampus were also found, but may have been induced by confounding effects of medication. The current study shows the potential of resting state analyses to differentiate between PTSD patients with versus without MDD and provides more insight in the neurobiological differences between these subgroups. These findings provide neurobiological markers for the presence of comorbid MDD in the context of PTSD and may potentially be targeted with treatment.

## Data availability

figshare: fMRI data of PTSD patients with and without comorbid Major Depressive Disorder, http://dx.doi.org/10.6084/m9.figshare.882837 ^62^
