# Supplementary material for: Altered functional connectivity in posttraumatic stress disorder with versus without comorbid major depressive disorder: a resting state fMRI study
Source: F1000Res. 2014 Apr 16;2:289. Originally published 2013 Dec 30. [Version 2] doi: 10.12688/f1000research.2-289.v2 (PMC4184309; doi:10.12688/f1000research.2-289.v2)
Supplement: Peer review history

# Peer review: Referee response for version 2

**DOI:** 10.5256/f1000research.4220.r4508

**Published:** 2014-06-09

**Authors:** Andrew Kemp

**Affiliations:** 1 Centre for Clinical and Epidemiologic Research, Hospital Universitário, University of São Paulo, São Paulo, Brazil; 2 Discipline of Psychiatry and School of Physiology, University of Sydney, NSW, Australia

I appreciate the authors detailed responses to my earlier comments. The reiterated manuscript makes a solid and welcome contribution to the literature. Well done!

I have read this submission. I believe that I have an appropriate level of expertise to confirm that it is of an acceptable scientific standard.

# Peer review: Referee response for version 2

**DOI:** 10.5256/f1000research.4220.r4792

**Published:** 2014-05-16

**Authors:** Onno Meijer

**Affiliations:** 1 Medical Pharmacology, Leiden University, Leiden, Netherlands

I think the authors did a decent job in reviewing the paper. I have no further comments to the content of the article.

I have read this submission. I believe that I have an appropriate level of expertise to confirm that it is of an acceptable scientific standard.

# Peer review: Referee response for version 1

**DOI:** 10.5256/f1000research.3319.r2919

**Published:** 2014-02-26

**Authors:** Onno Meijer

**Affiliations:** 1 Medical Pharmacology, Leiden University, Leiden, Netherlands

The paper shows convincing (to the technical non-specialist) differences in resting state functional connectivity between PTSD patients with or without comorbid major depression MDD.

There are clear effects on connectivity of the subgenual anterior cingulate cortex with the left ACC (increased with MDD) and with the thalamus (decreased with MDD).

The interpretations are hampered by the design, which ideally would be a 2 X 2, i.e. +/- MDD and +/- PTSD. Therefore sentences like 'we found that connectivity ... is more reduced in PTSD +MDD than in PTSD-MDD' cannot be made.

The authors determined that effects on connectivity of insula and hippocampus are related to PTSD symptom severity -  this should probably be indicated in figure 3 somehow, as this may end up being used as a graphic summary of the data.

I missed whether the authors looked at the use of medication as a factor that affected connectivity, as it may interact with the condition.

Second the authors, at correlation between connectivity (thalamus - sgACC) and symptom severity in the PTSD-MDD, group in a post hoc correlation. A similar effect is reported specifically for the PTSD+MDD group. It is unclear why the authors did not look at the correlation in all patients? What is the reason to now limit to a single group?

Interpretation: I do not see how on p.6 the authors suggest the cluster B symptoms would tilt the balance towards a PTSD only state, based on correlation, when table 1 clearly shows no differences between the groups for cluster B symptoms.

In terms of interpretation, I agree with the first reviewer that the statement in the abstract that the findings have impact on treatment development need to be substantiated. As markers? As targets? 

It also seems important whether or not PTSD and MDD *interact* in terms of connectivity, or whether the association of MDD and PTSD 'just' add up. For this a 2x2 design seems necessary, but the paper may benefit from actual meaning that is given to the data. Obviously, at present describing correlates is what is done in research like this, but an explicit sense of direction would be helpful to the non-specialist reader who is nevertheless interested in the subject matter.

**Minor remarks:**

- I am not certain that (as in: I do not think that) the second, long, sentence in the discussion accurately reflects the findings, please check.
- The term *however* in the third paragraph of the intro seems needlessly negative about previous research. 

I have read this submission. I believe that I have an appropriate level of expertise to confirm that it is of an acceptable scientific standard.

# Response

**Published:** 2014-04-07

**Authors:** Mitzy Kennis

**Affiliations:** University Medical Center Utrecht, Netherlands

We thank the reviewer for his valuable suggestions. See below our response to the comments.

**Design and interpretations:**

We agree with the reviewer that our study is limited by design, and lacks an MDD only group and a healthy control group. We acknowledge that we can therefore not clearly interpret the effects described here, with respect to the nature of either one of these disorders or the interaction of the two. As the reviewer suggests, we have attempted to be more cautious with our interpretations and elaborated on the implications (sense of direction, specific implications for treatment development) of the results in the discussion and abstract.

**Analyses:**

As proposed, additional analyses on a subsample of medication naive PTSD patients and PTSD patients that did not use benzodiazepines at least 48 hours prior to scanning were performed to investigate the effect of medication.

It was also questioned why we did not correlate the peak voxel of functional connectivity of the clusters of significant differences with all participants symptom scores, but only within groups. As also pointed out in our response to Andrew Kemp: a correlation analysis with the cluster of significant differences over all participants is inappropriate, since groups differ on both these values (functional connectivity cluster peak and PTSD severity (total CAPS score)) and therefore misleading correlations can be induced. To avoid this problem, within group correlations can be performed and seem more appropriate here. Finally, we have also added figures of the correlations with CAPS symptom clusters.

# Peer review: Referee response for version 1

**DOI:** 10.5256/f1000research.3319.r2918

**Published:** 2014-01-30

**Authors:** Andrew Kemp

**Affiliations:** 1 Centre for Clinical and Epidemiologic Research, Hospital Universitário, University of São Paulo, São Paulo, Brazil; 2 Discipline of Psychiatry and School of Physiology, University of Sydney, NSW, Australia

Thank you for the opportunity to review this interesting study on the impact of disorder comorbidity on resting state functional MRI. This is an important and under-studied area of research. I have a number of comments that may help to improve the manuscript.  

**Abstract: **

- The abstract is disappointing, dense and requires development. At present it is mostly comprised of statements relating to the findings obtained, rather than providing an interpretation of findings. Are all the findings reported key to the bottom-lines of the article? The authors need to add a brief interpretation after each of the KEY findings. What is the take home message; what are the bottom-lines? While the findings may indeed be “ *important for treatment development*”, it is unclear how. Please elaborate. 

 

**Introduction:**

- The first paragraph would benefit from a little more context on why comorbidity is important. For instance, studies have demonstrated that comorbidity between the mood and anxiety disorders increases risk for CVD and mortality ( Philips *et al.,* 2009). I would recommend the authors integrate this information to help build study rationale.
- While the rationale for focusing on subgenual ACC as a seed region in connectivity analysis is well-founded, I thought the rationale for focusing on the insula to be much weaker. I would have thought that the amygdala region would have been a more appropriate second choice, especially given the many past studies that have focused on the amygdala as a region of interest in patients with PTSD.  

 

**Methods:**

- re Participants section: who administered the psychiatric evaluation; were they psychiatrists or research assistants? How many people conducted the evaluations? How was the reliability and validity of this measurement across assessors determined? If research assistants, how much training did they receive? What was the value of the financial compensation received by participants? 
- re Data Acquisition: To what extent could this (incentivisation) have impacted on findings (i.e. group interaction) especially given the unrestricted participant instructions (i.e. relax and let the mind wander)? Was any questionnaire administered to assess mental state of participants during the resting state scan?

 

**Results:**

- I recommend the authors to review the article by Miller & Chapman (2001) on the problems associated with ANCOVA when participants are not randomly allocated to a group, as in the present study. The use of total CAPS scores as a covariate in post-hoc analysis of covariance is problematic. 
- One potential solution to this problem that the authors could consider is post-hoc correlational analysis. Although the authors ran a series of correlational analyses, these need to be conducted on all participants regardless of group, rather than by each group individually. The authors would need to determine the unique activations associated with the PTSD, with versus without depression, a contrast that is not associated with confounding variables (i.e. CAPS, positive affect).
- Another issue the authors need to consider is the impact of medication on their findings. While the authors note in their limitations that “ *future studies should investigate the effect of medication on the neurobiology of PTSD with or without MDD*”, the authors have a sufficient sample of medication naive participants on which sub-analysis could be run. 
- Error bars need to be added to Figure 3. 

 

**Discussion:**

- The authors link increases in subgenual-perigenual ACC connectivity in the PTSD+MDD group versus the PTSD-MDD group to differences in self-referential processing. This is an interesting point which I feel could be further elaborated (after of course confirming that these findings are still present after adjusting for confounds -see points I made regarding this issue under the results section). The same could be said for the discussion on the relationship between the disorders and the default mode network. 
- I also feel the discussion section needs to be further developed. Currently, much of the discussion restates the findings without more detail on what the differences might mean (especially in the first paragraph of the discussion). This makes the discussion section a little underwhelming. 

I have read this submission. I believe that I have an appropriate level of expertise to confirm that it is of an acceptable scientific standard, however I have significant reservations, as outlined above.

# Response

**Published:** 2014-04-07

**Authors:** Mitzy Kennis

**Affiliations:** University Medical Center Utrecht, Netherlands

We thank the reviewer for his valuable comments. See below our response to the comments per section of the manuscript.

**Abstract and introduction:**

We have attempted to make the abstract clearer, and added more context in both the abstract and introduction. We also acknowledge that the amygdala could have been another interesting seed  to investigate PTSD with and without comorbid MDD. However, since the two previous neuroimaging studies that investigated PTSD and MDD with task paradigms reported alterations in the insula ( Lanius *et al.*, 2007; Whalley *et al.*, 2009) while differences in the amygdala between PTSD and MDD patients were only reported by Whalley *et al.* (2009). Therefore, we argued that the insula is an appropriate candidate to investigate here.

**Methods:**

In order to address the concerns on the methods section, we provide more details on the administration of the interviews and financial compensation. Reliability and validity were qualitatively assessed by comparing interview scores and diagnosis between raters during interview training.

With respect to the resting state design, we do not expect any effects of the financial incentive on the pattern of functional connectivity. Every participant received a similar financial compensation for participating in the study. We assessed mental states of participants during resting state after the scan session, by asking whether they felt relaxed during the rest scan. Most participants confirmed feeling relaxed during the resting state scan, and ten participants reported having some difficulties to relax (five in each group). Furthermore, we investigated co-activation patterns of the subgenual ACC and insula. Although mind states are of influence on brain activation it is not expected that different mind states (e.g. individuals that tend to think of the incentive) directly alter the spatial co-activation patterns within these functional networks.

**Results:**

****

We agree with the reviewer that the use of ANOVA applied here is inappropriate. However, the suggested solution, a correlation analysis with the cluster of significant differences over all participants, is also inappropriate, since groups differ on both these values (functional connectivity cluster peak and PTSD severity (total CAPS score)). Therefore misleading correlations can be induced (see Lord’s Paradox; Lord, 1967).

Unfortunately, as Miller and Chapman (2001) have shown, there is no simple solution to “adjust” or “control” for differences between groups in non-randomized groups.

On the other hand, we agree with the reviewer that correlations can be explored in order to provide some insight in the relation between functional connectivity and symptom severity, but then *within* groups rather than over all participants (to avoid misleading correlations). Thus, we have explored correlations within PTSD+MDD and PTSD-MDD groups between symptom severity scores (total CAPS and reduced PA) and the peak voxels of significant differences. There were no significant correlations between PTSD severity and functional connectivity. This suggests that functional connectivity of the clusters of significant differences was not related to differences in total CAPS or reduced PA scores.

Additionally, for the correlations with the symptom clusters performed within groups (for the same reasons as described above), we plotted the data for all subjects and added a fitted line for the data for each group and for the whole group respectively (see Figure S2 and S3). This way, an objective overview of the data is presented and both whole group and subgroup correlations are visualized.

Finally, as requested we performed a post-hoc analysis to investigate the effect of medication by including medication naive PTSD patients and PTSD patients that did not use benzodiazepines at least 48 hours prior to scanning (PTSD+MDD n = 20, PTSD-MDD n = 15). This revealed similar results for the subgenual ACC seed, although the hippocampal cluster of difference in connectivity with the insula disappeared. This information has been added to the paper. Figure 3 now includes error bars.

**Discussion:**

We expanded the discussion section and added more detail on the interpretation of the results. Furthermore, we have nuanced the interpretation of the correlational analyses.
